# Supplementary material for: Chromothripsis during telomere crisis is independent of NHEJ, and consistent with a replicative origin
Source: Genome Res. 2019 May;29(5):737–49. doi: 10.1101/gr.240705.118 (PMC6499312; doi:10.1101/gr.240705.118)
Supplement: Supplemental Material [file supp_gr.240705.118_Supplemental_file_1.zip › contigs/annotated_contigs/DB57/contig.2.DB57_length_655_mean_cov_8.21984732824.docx]

**DB57_length_655_mean_cov_8.21984732824**

```
TTTCCAGAATACAGAGGTTTCCTTCATACTGAAGATTTGCTCTGACAAGTAATTCCCCCTCACAATCAACTTATCACTAATCAACTGAA
 >chr7:147364101-147364407 - E=2e-172
AACTAGAGTTTTCAGAAATTCTTCAGCTGTCTTCACATCAGCACTTGCAGACTCACCACTTACTTTCACATAACGTAATGAAAAACAGT

TCTTGAATCATTTAAACCACCCAGAGCTAGCAGTAAATTCAACATTGTAGTTGGGTCCAGCCTTTTCTTTCAACATTGCGAACAAACTT

CTTGCTTGGCTGTGATCATCATGGTGCTGGGAGGAATTC|TAGTATCAAAAATGATTGCCTTTGGTACTAGGCACACTTAGGTAGTACC
                                         >chr7:146824214-146824564 + E=5e-194
TAATATGTTCATTTCAGTGCAACTTGAAAACATTTTCCCAATAATTCTACTTTAGATTACTATTTCTTCTTTGTAGTTTTCTGATTCTT

TGGGATATATTTTTCCTCAATTTTCCTTTTACCTTCTTATATATGAACTGTGTGGCAAATAATACATATTCAGCTAACCATTATTAATA

ATAATTGATTTTATTGTAAATGAGGCCTTTGTTTTTTTTTTACCATCACCTCATAAACCACAATTTGTGTTTCCCCCCCTAAAAGTACT

TAAGCATTTAAGATGCCATAACACTAAATCATA
```
